# Socio-economic impacts of working horses in urban and peri-urban areas of the Cape Flats, South Africa

**DOI:** 10.4102/jsava.v91i0.2009

**Published:** 2020-04-16

**Authors:** Joanna N. de Klerk, Melvyn Quan, John D. Grewar

**Affiliations:** 1 Department of Biomedical Sciences, Institute of Tropical Medicine, Antwerp, Belgium; 2 Department of Veterinary Tropical Diseases, Faculty of Veterinary Science, University of Pretoria, Onderstepoort, South Africa; 3 Equine Health Fund, Wits Health Consortium, Johannesburg, South Africa

**Keywords:** cart horse, socio-economic, One Health, demographics, community, spatial epidemiology

## Abstract

In the Cape Flats townships, Cape Town, South Africa, there are more than 250 working cart horses. They serve the community with scrap metal and garden refuse removal, human transport and the selling of goods. A questionnaire was undertaken to understand the social and economic impacts of a horse and cart in the Cape Flats on individual owners and/or drivers, their households and the community. A mixture of classical quantitative questions combined with qualitative participatory technique questions were used. A total of 100 participants took part in the questionnaire, who cart with 163 horses between them. The majority (89%) identified the cart horse income as their primary income source. Apart from the participants, an additional 716 people were supported financially through this income, where the mean number of children supported was 2.9 (95% confidence interval [CI]: ±0.42) per interviewed participant. Scrap metal transportation was the most common work and the season (winter) had a negative impact on their ability to work. The spatial extent to which a cart horses work was determined and related back to the impact on the horse and participant of the survey. It was demonstrated that the cart horse industry had an impact not only on those who worked in the industry, but also on the surrounding residents, either through their work or through supporting others with their income. This study revealed that the concepts of ‘One Health’ and ‘Health in Social-Ecological Systems’, in action as horse and human health within the Cape Flats are closely intertwined.

## Introduction

According to Hart (1988), in the early 20th century when Cape Town, South Africa, was divided into districts, District Six had a rich economy in trading fresh fruit, vegetables, fish and bottles from the back of a cart in markets. It was very lucrative for the cart horse owner and horses were well looked after, only having to travel short distances, with small loads. As a result of apartheid in the 1970s, over 55 000 people were forcibly relocated to the Cape Flats townships from this area (Hart 1988:603–628). The markets were banned, which led to cart horse owners using their horses for collection of scrap metal as a means of making an income for themselves and their families. Recycling became increasingly popular at this time and larger companies started to look at reducing monetary inputs by recycling and as most dumpsites were located close to townships, reclaiming scrap metal became a viable income source (Benson & Vanqa-Mgijima 2010).

In 1976, Calvin Schwabe suggested a concept called ‘One Medicine’ which recognised the close-knit relationship between animals and humans in relation to their health, nutrition and livelihoods (Schwabe 1984:2). Since then, this concept has further developed to incorporate environmental health as an important factor and renamed ‘One Health’ (Zinsstag et al. 2011:148–156). More recently, socio-ecological systems have been integrated into the One Health approach to create a holistic multidisciplinary health system concept that recognises the links between not only human, animal and environmental health, but also social, economic, political and cultural aspects. Zinsstag et al. (2011:148–156) provisionally called this ‘health in social-ecological systems’ (HSES).

Within the Cape Flats, the use of cart horses influences the lives of their owners and the greater community through the economic impact of job creation, a social impact through transporting people and supporting the community, a human health impact through bettering their owners’ lives and an environmental impact through the removal and transporting of rubble, scrap and garden waste with a non-emission mode of transport. There are also animal health impacts seen in terms of health and welfare to the horses as a consequence of their work.

Equids are used throughout the world as a means of transport and income, although for many people in the community, their use is considered ‘backward and underdeveloped’ and associated with poverty (Fernando & Starkey 2004:459–508). Nevertheless, their use enables many people who have been marginalised to have the opportunity to be involved in various social and economic operations from which they otherwise would be excluded (Fernando & Starkey 2004:459–508). Horses or donkeys and carts can be highly efficient in both rural areas, where terrain is uneven and transport distances are short, and urban areas where distances are shorter than 30 km, loading and unloading time is proportionally higher than travel time and there are many collection and distribution points (Ramaswamy 1985:20–25).

In Africa, there are very few published studies that focus on the topic of socio-economic impacts of working equines, and the few that have been published have a focus on donkeys and not horses. The lack of studies may be because donkeys across many countries in Africa are considered to have a low status within the community, and therefore, research on them is a low priority (Swai & Bwanga 2008:1–97). This is counterintuitive, as it is recognised that draught animal power plays a vital role in development in developing countries and modernisation of it through surveys, studies, resource allocation, policy conception and action plans is beneficial (Ramaswamy 1985:20–25).

While the use of cart horses may enable the cart horse worker to climb out of a poverty trap through the provision of an income, it has been recognised that there is a knowledge gap in the literature regarding the implications of owning or working with a cart horse, with regard to labour and affordability, as well as the social demographics regarding the person working with the cart horse, who they support and where they stand in a family social structure (Wold, Tegegne & Yami 2004:79).

In the 1970s, alternative ways to pose questions were developed as it was recognised that data collection in poorly developed areas was not effective (Catley, Alders & Wood 2012:151–160; Chambers 1983). This was known as ‘participatory epidemiology’ (PE) and as it was developed, veterinary scientists began to use it more in their line of work (Catley 2000:702–714). The participatory epidemiology approach commonly includes semi-structured interviews that allow for discussion. Various question styles are used and often make use of visualisation techniques such as mapping, seasonal charts, proportional piling, ranking or scoring (Catley et al. 2012:151–160). Non-sampling errors deriving from poorly phrased or insensitive questions occur frequently, and therefore, testing the survey and adapting the questions accordingly are paramount (Pfeiffer 1996:159169). Proportional piling and ranking or scoring provide sound numerical data that can be analysed using conventional statistical methods (Catley et al. 2012:151–160).

From this study, we aimed to understand the social and economic impact the use of a horse and cart in the Cape Flats has on an individual, their household, the surrounding community and the horse itself and to understand the spatial extent to which the cart horses work through a questionnaire (Appendix 1).

## Materials and methods

### Data collection

A questionnaire form was created on the OpenDataKit (ODK) (https://opendatakit.org) Android platform to enable data collection on devices in the field over a period of two weeks in October 2017. This time was chosen because it was believed that more cart horses were active in fine weather compared to adverse weather conditions. A charity called ‘The Cart Horse Protection Association’ (CHPA), which works with the cart horses in the area, provided a complete list of all 189 owners and drivers of cart horses with reference to the areas in which they lived in the Cape Flats. Some owners had multiple horses. A total of 100 questionnaires were conducted between the CHPA centre in Epping and on field visits to outlying areas. A total of 100 surveys were decided on as it would be impossible to reach all 189 owners and drivers because of the nature of their work. This sample size would give a fair representation of the population. Pre-selecting participants were not possible, as most did not have means by which to contact them so participants were selected on a first-come-first-served basis. Monitoring of proportional surveying based on five grouped locations in which the CHPA worked was performed to ensure that the 100 surveys were spatially representative. Therefore, after several days, participants who were located in areas where many people had already participated in the survey were turned away. By the end of the survey period, this created a sample that was proportionately accurate of the entire population. Assistants of drivers, known as guards, were not allowed to participate as they were not directly in charge of the horse or earning the income from the work. To remove answer bias, participants were informed verbally that interviewers were not associated with the CHPA and CHPA employees were not physically present during any of the interviews. All participants received an incentive of a R100.00 (South African Rand) food voucher to encourage willing participation. All participants gave informed consent.

The questionnaire was delivered in English and translated to Afrikaans if necessary. It was completed in ODK by the interviewer.

Both PE styles of questions and classical styles of questions were used to attempt to get accurate responses from a population where education levels were basic as recommended by Upjohn et al. (2003:313–320). Binary questions and frequency questions were asked, as well as more visual and interactive PE techniques such as proportional piling to resemble percentages of time worked in certain fields, choosing between sets of pictures and drawing on a map the neighbourhoods worked in. There were places for optional comments to be added throughout. At the end of the survey, there was a question for the interviewer to answer. This question was to assign a score, on a scale of 1–10, on how much the participant appeared to approximate their answers.

### Data analysis

Data were extracted into Microsoft Excel and then imported into R Studio (R Core Team 2017) for analysis (Table 1-A2, Appendix 2). Tests of association included Pearson χ^2^ and one-way tests. A significance level was set so that the result would be significant if *p* < 0.05. QGIS (QGIS Development Team 2016) was used for geospatial analysis and visualisation. Each neighbourhood polygon in the Cape Flats was coded. Neighbourhoods were digitised manually using the latest available 1:50 000 base layer maps. Multi-polygons per interview were recorded in lists by visually comparing the photographed map of areas worked taken during the interview. Points were marked where the horses were stabled and digitised per interview. The distance travelled to work was measured by the point at which the horses were stabled to the centroid of the polygon of the area worked by using SQL queries in a PostGIS-enabled PostgreSQL database.

### Ethical considerations

Ethical clearance to conduct the study was obtained from the University of Pretoria’s Department of Humanities (Ref no. 356/2017). Therefore, this research has been performed in accordance with the ethical standards laid down in the 1964 Declaration of Helsinki and its later amendments.

## Results

### Demographics

Out of 100 participants, 98 were men and two were women. Between the participants, there were an associated 163 horses representing approximately 57% of the known population. The mean number of cart horses worked with by a single participant was 1.7 (95% confidence interval [CI]: 1.5–1.9), with a range of 1–6. This did not include young unbroken (generally under three years old) horses which may become cart horses in the future. Out of 100 participants, 716 people were either partially or fully supported financially, not including themselves. The mean number of children supported was 2.9 (95% CI: 2.5–3.3), the mean number of family adults supported was 2.2 (95% CI: 1.8–2.6), the mean number of employees supported was 1.0 (95% CI: 0.8–1.2) and the mean number of other non-related people supported was 1.1 (95% CI: 0.7–1.5) per person interviewed. Other non-related people included 24 friends, 50 people in the community, 17 people in the community in return for once off work and 14 unspecified. Out of 89 drivers or owners who also drove their own horses, 71 (79.8%) worked with guards to aid them with loading goods and handling the horse, for at least one of the horses they drove.

Classification of the people who participated in the survey showed that 13.5% were the owners of the cart horse, 44.2% were the drivers and 42.3% were the owners as well as the drivers. The size of the cart horses, described in hands (hh), showed a distribution of 20.1% small ponies between 12 and 13 hh, 44% large ponies between 13 and 14 hh and 35.8% horses between 14 and 15 hh. Other horse demographics were not collected. The primary caretakers of the horses were 55.2% owners, 36.8% drivers, 1.8% children related to the participant of the survey, 1.2% other adult family members of the participant of the survey and 4.9% an employee of the participant of the survey.

### Economic impacts

The majority (89%) of the participants identified working with the horses as their main source of income. Other sources of income included other lines of work, especially if the participant was the owner of the horse and not a driver, government grants and pensions and one man identified the horse as not a source of income, but rather a means of transport for his children travelling to and from school.

**FIGURE 1:**
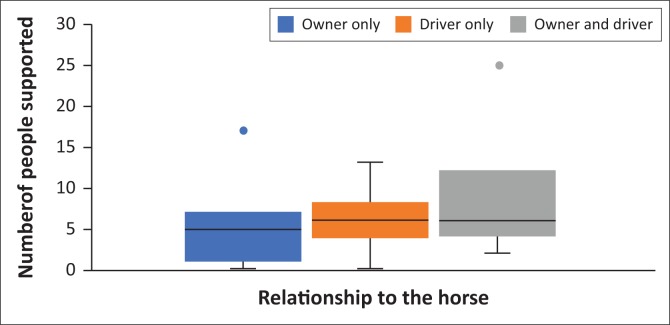
Boxplots to show the relationship between the participant’s relationship to their horse and the number of people they support. Boxes represent the range of number of people supported between the first and third quartiles with the median indicated by the bar running through the box. The whiskers represent the minimum (below) and maximum (above) number of people and outlier results are indicated by dots separate to the whiskers.

The relationship with a horse, of which the options were owner only, driver only and both owner and driver, was compared to how many people were supported based on their horse-related income only and there was a significant difference. Figure 1 shows that participants of the survey, who were both owners and drivers of the horses they worked with, supported significantly more people than those who were just owners, and slightly more than those who were just drivers. Figure 1 also shows that those who were only drivers supported more people than those who were only owners.

The distribution of earnings on a normal working day from horse-related income (before deductions) showed that 60% of participants earned R0.00 – R300.00 per day, 36% earned between R300.00 and R600.00 per day and 4% earned R600.00 – R900.00 per day. No participant earned over R900.00 on a normal day although some commented that on a good day they might earn up to R1000.00 if they collected a car body as scrap metal. Fifty-eight participants could give an exact figure of what they earn on a normal working day: the mean average earned was R287.07 (95% CI: 257.76–316.38).

**FIGURE 2:**
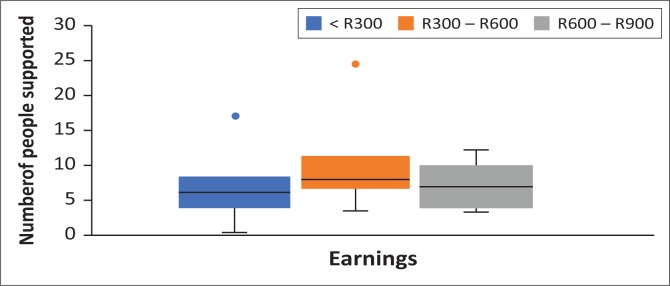
Boxplots to show the relationship between the daily earnings of the participant and the number of people they support. No participant earned more than R900.00 per day. Boxes represent the range of number of people supported between the first and third quartiles with the median indicated by the bar running through the box. The whiskers represent the minimum (below) and maximum (above) number of people and outlier results are indicated by dots separate to the whiskers.

The relationship with the horse and its association with daily earnings from the cart horse was evaluated and it was found that even though the type of relationship with the horse influenced how many people were supported, it did not influence the daily income earned (*χ*^2^ [2, *N* = 100] = 0.193, *p* < 0.05). When the daily earnings generated with the number of people supported were evaluated, there was a significant difference between daily earnings categories and number of people supported in each category. Figure 2 shows that those earning R300.00 – R600.00 per day supported more people than those earning less than R300.00 per day. As there were very few survey participants earning between R600.00 and R900.00 (*N* = 4), the outcome that they supported fewer than those in the R300.00 – R600.00 per day category may not be a reliable result.

Scrap metal transportation was the most popular type of work with a mean percentage of 46.6% of the work carried out by all participants. Garden refuse removal was second most popular at 32.3%. Other types of work, such as selling things from the back of the cart, accounted for 14.2% of work. Of all the participants (*N* = 39) who indicated that they used the cart for other work, five said they sold mattresses, six said they sold furniture, eight said they sold food, eight said they moved household items, two said they moved wood or building materials, six transported other items that needed dumping and four did not specify what their other work was. The least common type of work was transporting people, with a mean of 6.8% of the work performed. Each type of work was ranked 1–4: 1 representing the type of work they carried out most often and 4 being the least often. Table 1 outlines the median rank for each earning category. Type of work did not appear to influence earnings as scrap metal transportation was close to rank 1 for all the earnings categories and there was not an association between type of work and income category.

**TABLE 1:** Median rank of types of work versus income category where the closest to 1 was the top rank and the closest to 4 was the lowest rank.

Earnings	Scrap metal	Garden refuse	Transport	Other†
< R300.00 per day	2.0	2.0	4.0	3.5
R300.00 – R600.00 per day	1.0	2.0	4.0	4.0
R600.00 – R900.00 per day	1.5	2.5	2.0	3.0

† , Other work included selling mattresses, furniture and food, moving household items, wood or building materials and transporting other items for dumping.

Out of the drivers who did not own the horse, 92% confirmed that they had to pay the owner either a fixed sum or a cut of their earnings to use the horse. After that payment, 46% then had to use the remaining money to pay towards the horses’ shoes, and 35% had to pay towards the horses’ feed. More than half of participants who were only drivers (54%) indicated that the owner paid for all things related to the horses’ care. Finally, 77% of horses were driven with a guard, rather than alone, and many drivers commented that the remaining money from the day after deductions was split equally between themselves and the guard.

### Seasonal and social impacts

Seasons had a major impact on the ability to work according to 82% of participants, of which 50% of these indicated that they worked less in winter and 32% cited that the rain was a factor, as the horses could sometimes not work and items on the back of the cart would get damaged. However, the summer also posed problems as 16% of these participants were concerned about the heat. Of the horses that were used, 13.5% were used in all weathers regardless, 47.9% were used sometimes in inclement weather depending on how bad it was and 38.7% were never used in bad weather.

The areas where the cart horses were stabled had an impact, as six participants commented that gang violence stopped their combined 15 horses from being able to get out and go to work.

### Spatial analysis

**FIGURE 3:**
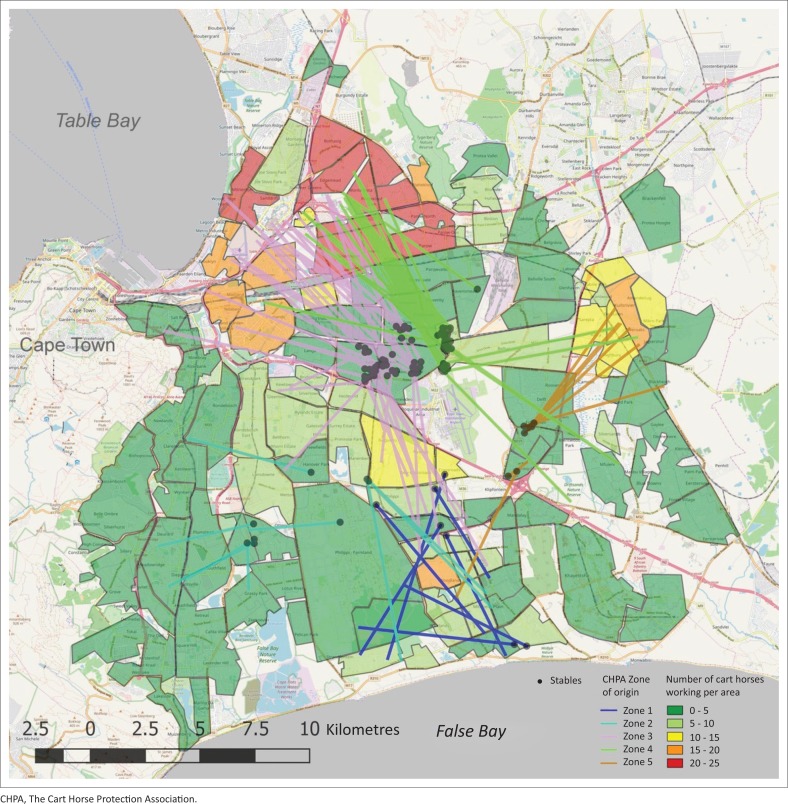
A map to represent the number of cart horses regularly working in each neighbourhood. Dots indicate stable locations. Areas are coloured on a green to red scale highlighting the number of horses working per area. Also, the median angle of direction and mean distance travelled from each stable point are demonstrated and colour coded for each of the five areas.

Figure 3 shows the number of cart horses that worked routinely in each coded neighbourhood, as well as the location of the stables where cart horses are stabled at night and the mean distance traveled at the median angle of direction from each stable.

The distance travelled from the stable to the centroid point of each neighbourhood was calculated. The minimum distance travelled from the stable to the closest neighbourhoods’ centroid point was 0.25 km, the maximum distance from the stable to the furthest neighbourhoods’ centroid point was 17.6 km and the mean distance to the neighbourhoods visited was 7.2 km (95% CI: 3.6 km – 10.8 km). As it was impossible to measure the total daily distance travelled, it was assumed that the greater the average and maximum distances to each neighbourhood, the greater the total daily travel.

There was a significant difference between daily earnings and distance travelled for both the average distance and maximum distance. The further the distance travelled by the cart horse to neighbourhoods, both on average and maximum distance, the higher the daily earnings.

### Accuracy of the results

At the end of the survey, a question was asked to the surveyor about their opinion on the level of approximation from the participant. The mean estimation score was 7.42/10 (95% CI: 7.2–7.64).

## Discussion

There are over 111.6 million equines in the world, of which 22.7 million are located in what is considered to be the 31 least developed countries in the world (FAO 2014), which is a disproportionately higher number of equines in these developing countries. In these countries, the equine’s lives are mainly spent working (Mekuria & Abebe 2010:3) and the use of these equines will have a great impact on the livelihoods of their owners in these resource-poor areas.

The only published data on the socio-economic impacts of working equines in South Africa were written by Wells and Krecek (2001:37–43). They undertook a small survey of 21 owners of 104 donkeys and mules in the North-West Province of South Africa over one year between 1995 and 1996. The equids were used for ploughing, carrying packs, carrying loads by cart and transporting people by cart. This study investigated socio-economic, health and management aspects of these working equines. It was shown that equines played a large role in the community. Almost a third of owners (30%) derived their main income from the equines, while another 40% derived their main income from the farming which could not be performed without the equines, and 70% of owners allowed other family members to borrow them if needed. This study was an insightful primary study into some factors relating to owning working equines in South Africa, especially focussed on a rural area, but there is no published literature looking into the use of working equines in urban areas in South Africa.

In the rest of Africa, there have been minimal published studies looking at the use of cart horses; however, working donkeys are the focus of several studies. In a study in Northern Tanzania, it was reported that it is customary in the study area for a donkey owner to lend a donkey, rather than renting it out, to someone to aid in transport of foodstuffs, water and other household goods via pack or cart, and therefore, provide an important social impact within the families and surrounding communities (Swai & Bwanga 2008:1–97). This is in contrast to the industry in the Cape Flats, where renting out equines is a way for owners to make money.

Our survey investigated whether the working horses in the developing region of Cape Town, the Cape Flats, had a socio-economic impact on their owners, drivers and surrounding community. An interview with the Head of Fundraising and Development of the CHPA revealed that this work was mainly performed by men, with 98 of the participants being men, potentially because women find it easier to source jobs as domestic workers or nannies in these communities (M. White, Cart Horse Protection Association, pers. comm., 23 Oct. 2017).

The average income figure of R287.00 per day per participant does not take into account paying for shoes or feed for the horse, a driver paying the owner for the use of the horse or hiring a guard to help with the work. In areas where stables are densely located, the average yearly household income in Elsies Rivier was R38 201.00 – R76 400.00 and R19 601.00 – R38 200.00 in Gugulethu and Delft (Stats SA 2017). If the mid-range figure of R38 200.00 was used to calculate a daily income, based on working 5 days per week, 52 weeks of the year, the daily income would be R146.93. This is about half the average daily income of the cart horse workers; however, if the expenses are subtracted, it is likely to work out at a similar figure. Nevertheless, Statistics South Africa reports an average household income, not a personal income, and if partners of the cart horse owners also worked, this could put them in a higher income band compared to the rest of their neighbourhood. This salary is still small, but considering that unemployment rates in these areas are 10.2% in Elsies Rivier, 16.6% in Delft and 19.3% in Gugulethu (Stats SA 2017), these residents at least have a form of income, which evidently goes towards supporting a further number of people. Those participating in the questionnaire which both owned and drove their own horse, and those which on average travelled further were able to support more people and earn more money, respectively. This is maybe because they would have access to more clients on a daily basis, as well as not having to pay another owner to use the horse, taking a cut out of the salary.

Several questions were asked regarding external factors influencing work, which included questions about the season, weather and scrap metal prices.

These were asked in three ways: simple yes or no answers, that is, ‘Do seasons/weather affect the type or frequency of your work?’; open questions, that is, ‘How do seasons/weather affect the type or frequency of work?’ and multiple-choice questions, that is, ‘Does bad weather stop you from using this horse? Options were: a. I’ll use it in all weather, b. It depends how bad the weather is, c. I will never use it in bad weather’. Asking the question in three ways allowed a full picture to be created about the impact of seasons and weather on the cart horse and the people who work with them. A comment was made that the roads become dangerous in the wet conditions, and very few cart horse owners will take their horses out in rain. For those drivers who do, the CHPA stops them when out on patrols. Another comment also mentioned that rain will ruin items being sold off of the back of the cart. According to the Cape Town weather station and data compiled by the World Meteorological Organization, between 1969 and 1990, Cape Town received on average 70 days of precipitation per year (Yr 2017). This eliminates 19.2% of potential workable days of the year. Seasons were also impactful on types of work, with more garden refuse available in the summer. However, there were varying responses about the winter, including that participants worked with more scrap metal transportation in the winter because of less garden refuse being available, but also that they worked with less scrap metal in the winter because of the greater demand for it within the cart horse community and therefore less availability.

The distance from the stables to the neighbourhoods worked in varied greatly within the data, ranging in a straight line from 0.25 km to 17.6 km, and the mean distance travelled was 7.2 km. It is more realistic that the cart horses travelled in a circular route on a daily basis, and therefore, these figures are not representative of the true distance the horse travels in 1 day. It does provide a parameter that can be compared easily and it is likely to be related to the entire distance travelled on the circular route. Figure 3 shows the median angle of travel, as well as the mean distance, and many of the larger distances travel south towards Gugulethu and Mitchells Plain, and east towards Kuils Rivier. These places can be seen from the map to be much less dense for cart horse work compared to the areas in the north, such as Bothasig and Edgemead. Further investigation is required to consider whether further distances travelled lead to higher income, as it could be that there is more work available in those particular neighbourhoods because of a smaller density of horses working there, or that those areas might be more affluent than others. Even though it appears that the size of the spatial extent in which a cart horse works has a direct impact on how successful that cart horse owner or driver is, the results may be confounded by the number of carts that work in those areas, or the affluence of the areas. This requires further study using various other cart horse populations to ascertain the effect of distances travelled on a successful business as, currently, no other publications have studied this, and Cape Town provides too many confounders to reliably conclude either way. Also, by using route distances rather than straight line distances, this will provide more accurate data on how far the horses truly travel. This could be performed by mapping routes using a global positioning system (GPS) tracker on carts.

## Conclusion

The cart horse industry in the Cape Flats is a truly unique impactful industry not only in the Cape Flats areas, but also expanding into many surrounding neighbourhoods in the greater City of Cape Town. Without these horses and carts, the scrap metal industry and garden refuse removal industry would look very different, probably supporting far fewer families and being transported by vehicles with much higher carbon emissions.

The cart horses clearly have enormous social and economic impacts on both the individual, their families and the surrounding communities. This research has discovered a great network of people who are supported by the income from the cart horses, through directly working with the horses, being employed by owners or drivers, or having a relationship with them. Not only do the horses provide a monetary impact on these people, working allows people to move out of unemployment, which can otherwise lead to street crime and gang violence in these crime-rich areas. The spatial extent in which these horses work is not limited to the Cape Flats, where many are stabled, but instead covers many neighbourhoods in the City of Cape Town, impacting the lives of thousands of residents.

This study highlights a clear example in which a society has addressed their needs through a One Health approach. The interconnected nature of human, animal and environment health encouraged the collaboration of people from different disciplines to overcome the challenges faced by them in their society.

Without the cart horse industry in Cape Town, it would be a very different place, particularly in the Cape Flats where unemployment rates are high.

## Appendix 1: Mate rial – Survey

1. I have read and understood the consent form (Provided before the questionnaire).

### Background

2. How many horses do you work with?
3. What relationship(s) do you have with your horse(s)?
  - Owner
  - Driver
  - Owner and driver
4. Is your income from the horse(s) your primary income?

### Benefiters

5. How many children under 18 (family) benefit directly from your horse income?
6. How many adults (family) benefit directly from your horse income?
7. How many employees (domestic worker, shepherds, grooms, etc.) benefit directly from your horse income?
8. How many other (non-family) adults benefit directly from your horse income?
9. Comments if other adults benefit.

### Work proportions (only answered from drivers)

Four pots and 100 pebbles are provided for participants to split the pebbles up to resemble each category below in questions 10–13.

10. What proportion of the time do you use a horse for scrap metal work?
11. What proportion of the time do you use a horse for garden refuse removal?
12. What proportion of the time do you use a horse for transporting people?
13. What proportion of the time do you use a horse for other work not mentioned?
14. Comments on other work.

### External factors

15. Do scrap metal prices affect the type or frequency of your work?
  - How?
16. Do seasons affect the type or frequency of your work?
  - How?
17. What is the height of your horse(s) which you use?
  - Small pony (12 hh–13 hh)
  - Large pony (13 hh–14 hh)
  - Horse (14 hh–15 hh)
18. From the pictures, which carts do you consider to look ok to pull? (show four pictures of horses and carts combinations)
  - 1
  - 2
  - 3
  - 4
  - 5
19. If there is no traffic slowing you down, how fast would you travel?
  - Walk
  - Trot
  - Canter
  - Gallop

### Horse questions (repeat whole set for each horse owned)

20. How do you identify this horse?
21. What relationship do you have with this specific horse?
  - Owner
  - Driver
  - Owner and driver
22. Did you buy the horse? (Owner)
23. Do you pay to use the horse? (Driver)
24. What are you responsible for paying for? (Driver)
  - Shoes
  - Feed
  - Neither of the above
25. Do you use a guard to recruit other people when working with this horse? (Driver)
26. How many days per week does this horse work?
27. How frequently does this horse have problems stopping it from being able to work?
  - Never
  - Rarely
  - Sometimes
  - Regularly
  - Always
28. In the last year has this horse had problems because of ill health?
29. In the last year has this horse had problems because of injury?
30. In the last year has this horse had problems because of exhaustion?
31. In the last year has this horse had problems because of shoeing problems?
32. In the last year has this horse had problems because of cart or harness-related issues?
33. How often will bad weather stop you from using this horse?
  - I will use it in all weathers
  - It depends how bad the weather is
  - I will never use it in bad weather
34. In the last year has this horse had problems because of other reasons not mentioned?
35. Comments on other reasons.
36. Who is most often in charge of looking after this horse? (feeding, giving water, shoeing, etc.).
  - Owner
  - Driver
  - Children under 18 years old (family)
  - Adults (family)
  - Employees (domestic worker, grooms, shepherds, etc.)

### Communications

37. Who do you call first if there is an injury or illness of the horse that you cannot treat?
  - Cart Horse Protection Association
  - A friend or family member
  - Another cart horse owner
  - A veterinarian
  - Other
38. Comments on other communications.

### Other

39. Circle the areas on the map where you usually work and place a cross where the horse(s) is stabled.
40. On average, how much do you earn on 1 day from your horse(s)?
  - R0–300
  - R300–600
  - R600–900
  - R900+
  - 40.1. Comments if exact figures discussed.
41. Interviewer comments on level of estimation throughout survey.

## Appendix 2: Statistical tests

**TABLE 1-A2:** Statistical test results computed using R.

Comparison	Test used	Result	Significance	Confidence level (%)
Relationship to horse and number of people supported	One-way test	0.005	Significant	95
Relationship to horse and daily earnings	Pearson χ^2^	0.193	Not significant	95
Number of people supported to daily earnings	One-way test	0.018	Significant	95
Earning category to average distance travelled	One-way test	0.019	Significant	95
Earning category to maximum distance travelled	One-way test	0.003	Significant	95

## References

[CIT0001] Benson K. & Vanqa-Mgijima N., 2010, ‘Organizing on the streets: A study of reclaimers in the streets of Cape Town’, *Women in Informal Employment: Globalizing and Organizing*, viewed 01 August 2017, from http://www.wiego.org/sites/default/files/publications/files/Benson-Vanga-Mgijima_WIEGO_OB4.pdf.

[CIT0002] Catley A., Alders R.G. & Wood J.L., 2012, ‘Participatory epidemiology: Approaches, methods, experiences’, *The Veterinary Journal* 191, 151–160. 10.1016/j.tvjl.2011.03.010 21856195

[CIT0003] Catley A., 2000, ‘The use of participatory appraisal by veterinarians in Africa’, *Revue scientifique et technique (International Office of Epizootics)* 19, 702–714. 10.20506/rst.19.3.1239 11107613

[CIT0004] Chambers R., 1983, *Rural development: Putting the last first*, Longman Scientific and Technical, New York, NY.

[CIT0005] FAO, 2014, *FAOSTAT*, viewed 14 December 2017, from http://www.fao.org/faostat/en/#data/QA.

[CIT0006] Fernando P. & Starkey P., 2004, ‘Donkeys and development: Socio-economic aspects of donkey use in Africa’, in Starkey P. & Fielding D. (eds.), *Donkeys, people and development. A resource book in the animal traction network for Eastern and Southern Africa*, pp. 459–508, ACP-EU Technical Centre for Agricultural and Rural Cooperation, Wageningham.

[CIT0007] Hart D.M., 1988, ‘Political manipulation of urban space: The razing of District Six, Cape Town’, *Urban Geography* 9(6), 603–628. 10.2747/0272-3638.9.6.603

[CIT0008] Mekuria S. & Abebe R., 2010, ‘Observation on major welfare problems of equine in Meskan district, Southern Ethiopia’, *Livestock Research for Rural Development* 22(3), 1–15.

[CIT0009] Pfeiffer D.U., 1996, ‘Survey data collection methods – Questionnaires and interviews’, in Zimmermann W., Pfeiffer D.U. & Zessin K.H. (eds.), *Primary animal health activities in southern africa. deutsche stiftung internationale entwicklung*, International Seminar on Primary Animal Health Activities in Southern Africa, MZUZU, Malawi, February 02, 1996, pp. 159–169.

[CIT0010] QGIS Development Team, 2016, *QGIS Geographic Information System*, computer software, Open Source Geospatial Foundation Project, Chicago.

[CIT0011] R Core Team, 2017, *R: A language and environment for statistical computing*, computer software, R Foundation for Statistical Computing, Vienna.

[CIT0012] Ramaswamy N., 1985, ‘Draught animal power–socio-economic factors’, in Copland JW., (ed.), Draught Animal Power for Production: Proceedings of an international workshop held at James Cook University, Townsville, Qld, Australia, July 10–16, 1985, pp. 20–25.

[CIT0013] Schwabe C., 1984, *Veterinary medicine and human health*, p. 2, Williams and Wilkins, Baltimore, MD.

[CIT0014] Stats SA, 2017, *City of Cape Town Statistics*, viewed 19 October 2017, from http://www.statssa.gov.za/?page_id=993&id=city-of-cape-town-municipality.

[CIT0015] Swai E. & Bwanga S., 2008, ‘Donkey keeping in northern Tanzania: Socio-economic roles and reported husbandry and health constraints’, *Livestock Farming* 20(5), 1–97.

[CIT0016] Upjohn M., Attwood G., Lerotholi T., Pfeiffer D. & Verheyen K., 2013, ‘Quantitative versus qualitative approaches: A comparison of two research methods applied to identification of key health issues for working horses in Lesotho’, *Preventative Veterinary Medicine* 108(4), 313–320. 10.1016/j.prevetmed.2012.11.008 23419786

[CIT0017] Wells D. & Krecek R., 2001, ‘Socioeconomic, health and management aspects of working donkeys in Moretele 1, North West Province, South Africa’, *Journal of the South African Veterinary Association* 72(1), 37–43. 10.4102/jsava.v72i1.607 11563717

[CIT0018] Wold A.G., Tegegne A. & Yami A., 2004, ‘Research needs of donkey utilisation in Ethiopia,’ in Starkey P. & Fielding D. (eds.), *Donkeys, people and development. A resource book in the animal traction network for Eastern and Southern Africa*, p. 79, ACP-EU Technical Centre for Agricultural and Rural Cooperation, Wageningham.

[CIT0019] YR, 2017, *Weather statistics for Cape Town, Western Cape (South Africa)*, viewed 06 January 2018, from https://www.yr.no/place/South_Africa/Western_Cape/Cape_Town/statistics.html.

[CIT0020] Zinsstag J., Schelling E., Waltner-Toews D. & Tanner M., 2011, ‘From “one medicine” to “one health” and systemic approaches to health and well-being’, *Preventative Veterinary Medicine* 101(3–4), 148–156. 10.1016/j.prevetmed.2010.07.003 PMC3145159 20832879

